# A Mix of Natural Bioactive Compounds Reduces Fat Accumulation and Modulates Gene Expression in the Adipose Tissue of Obese Rats Fed a Cafeteria Diet

**DOI:** 10.3390/nu12113251

**Published:** 2020-10-23

**Authors:** Albert Gibert-Ramos, Miguel Z. Martín-González, Anna Crescenti, M. Josepa Salvadó

**Affiliations:** 1 Department of Biochemistry and Biotechnology, Nutrigenomics Research Group, Universitat Rovira i Virgili, 43007 Tarragona, Spain; miguel.martin@estudiants.urv.cat (M.Z.M.-G.); mariajosepa.salvado@urv.cat (M.J.S.); 2 Eurecat, Centre Tecnològic de Catalunya, Unitat de Nutrició i Salut, 43204 Reus, Spain

**Keywords:** obesity, white adipose tissue, polyphenol, conjugated linoleic acid, GSPE, anthocyanin

## Abstract

Scientists are focusing on bioactive ingredients to counteract obesity. We evaluated whether a mix containing grape seed proanthocyanidin extract (GSPE), anthocyanins, conjugated linoleic acid (CLA), and chicken feet hydrolysate (CFH) could reduce body fat mass and also determined which mechanisms in the white adipose tissue (WAT) and the brown adipose tissue (BAT) were affected by the treatment. The mix or vehicle (VH) were administered for three weeks to obese rats fed a cafeteria (CAF) diet. Biometric measures, indirect calorimetry, and gene expression in WAT and BAT were analyzed as was the histology of the inguinal WAT (IWAT). The individual compounds were also tested in the 3T3-L1 cell line. The mix treatment resulted in a significant 15% reduction in fat (25.01 ± 0.91 g) compared to VH treatment (21.19 ± 1.59 g), and the calorimetry results indicated a significant increase in energy expenditure and fat oxidation. We observed a significant downregulation of *Fasn* mRNA and an upregulation of *Atgl* and *Hsl* mRNA in adipose depots in the group treated with the mix. The IWAT showed a tendency of reduction in the number of adipocytes, although no differences in the total adipocyte area were found. GSPE and anthocyanins modulated the lipid content and downregulated the gene and protein levels of *Fasn* compared to the untreated group in 3T3-L1 cells. In conclusion, this mix is a promising treatment against obesity, reducing the WAT of obese rats fed a CAF diet, increasing energy expenditure and fat oxidation, and modifying the expression of genes involved in lipid metabolism of the adipose tissue.

## 1. Introduction

The number of obesity cases worldwide is increasing yearly. In 2016, 39% of the adult population were overweight and 13% were obese [1]. Obesity is characterized by an abnormal accumulation of fat caused by an imbalance between caloric intake and expenditure, with serious implications for health [1]. Weight loss accompanied by body fat reduction has a beneficial impact on overall health in obese individuals, including a reduction in the risk of cardiovascular diseases [2] and decreased insulin resistance [3], among others.

The white adipose tissue (WAT) is the principal organ affected in obesity, increasing the mass (hypertrophy) and number (hyperplasia) of adipocytes through the incorporation of triglycerides (TAG) into its cytoplasm [4]. It has been shown that the WAT tends to be hypertrophic in obesity, promoting insulin resistance and hypertriglyceridemia and leading to the accumulation of lipids in peripheral tissues [5]. Although adipocytes are the principal cells in WAT, it also includes a heterogeneous cell population called the stromal vascular fraction (SVF) [6]. Adipocytes have been extensively studied in vitro in the murine 3T3-L1 cell line, which is a good model to study the effects of particular compounds on adipocyte differentiation [7,8,9].

In contrast to the WAT, the brown adipose tissue (BAT) specializes in energy expenditure (EE) due to the high number of mitochondria in its cytoplasm that contain uncoupling protein 1 (UCP1), which dissipates the proton gradient inside the mitochondria, thus generating heat instead of ATP [10]. For this reason, it has drawn the attention of scientists as a target for obesity treatment.

Different approaches can be used to tackle obesity and reduce the size of the adipose tissue, such as caloric restriction or the use of weight-loss drugs. However, these practices have limited efficacy due to side effects or limited long-term success [11,12]. For this reason, researchers are studying other methods that could improve health in obese individuals using natural bioactive compounds, such as polyphenols, fatty acids, or peptides [13].

Our research group has designed [14] a mix of natural compounds that have individually shown to exert certain beneficial effects against different complications associated with obesity. This mix consists of the combination of a grape seed proanthocyanidin extract (GSPE), anthocyanins extracted from bilberry and blackcurrant, conjugated linoleic acid (CLA), and chicken feet hydrolysate (CFH). GSPE, which consists of several polyphenolic flavonoids, has been reported to reduce the weight of different WAT depots in hamsters fed a high-fat diet (HFD) [15]. However, in cafeteria-fed rats, GSPE reduced adipocyte size and increased the number of adipocytes without affecting the weight of the body fat [16]. Anthocyanins have been reported to reduce the weight of the adipose tissue and suppress fatty acid synthesis genes in obese mice [17]. CLA has been shown to increase EE and the browning of the WAT of obese mice [18]. Furthermore, CFH has been shown to reduce blood pressure in hypertensive rats [19,20]. Even though GSPE [21], anthocyanins [22], and CLA [23] have all shown promising effects on the adipose tissue in obesity, the results of previous studies have been varied and inconclusive and further research is needed. On the other hand, CFH has been studied as an antihypertensive peptide, and there are no reported effects on the adipose tissue. In this sense, the purpose of its addition into the mix is mainly for its effects on hypertension. Altogether, we hypothesized that supplementation with a mix of GSPE, anthocyanins, CLA, and CFH ingredients could have an advantageous effect on the treatment of obesity and related diseases compared to the individual ingredients due to complementarity or synergy of the effects of the ingredients in different processes related to the development of obesity. Our research group has researched this mix of ingredients through different studies, each focusing on different organs or ailments related to obesity. The aim of this particular study was to evaluate the ability of the mix to reduce body fat of obese Wistar rats by modifying the metabolism of WAT and BAT tissues.

## 2. Materials and Methods

### 2.1. Mix Composition

GSPE was kindly provided by Les Dérives Résiniques et Terpéniques (Dax, France), and its composition has been previously characterized by Margalef et al. [24]. Anthocyanin extract was kindly provided by the Biolink Group (Sandnes, Norway), and it is based on anthocyanins extracted from bilberries (*Vaccinium myrtillus*) and blackcurrant (*Ribes nigrum*); its composition has been described by Qin et al. [25]. The CLA used was Tonalin^®^ CLA (Cognis, Illertissen, Germany), and it is principally composed of CLA isomers c9, t11 and t10, c12. Specifications of the composition were provided by the manufacturer and are described in the Table S1. The manufacturing method and composition of CFH are described by Bravo et al. [26].

### 2.2. Animals and Treatments

**Figure 1:**
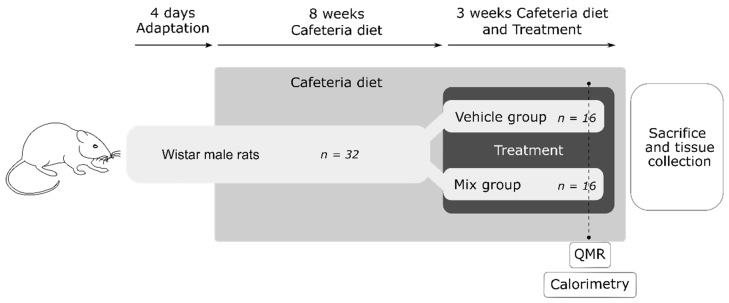
Experimental design of the animal study. A total of 32 Wistar male rats were adapted to the housing conditions for four days. Animals were fed a cafeteria diet *ad libitum* for 11 weeks. The body weight and food intake of the animals were recorded once each week. After eight weeks of cafeteria diet, animals were randomly distributed in two groups (*n* = 16) and orally supplemented with a mix (mix group) of natural ingredients, composed of 25 mg grape seed proanthocyanidin extract (GSPE)/kg body weight, 100 mg conjugated linoleic acid (CLA)/kg body weight, 100 mg anthocyanins/kg body weight, and 55 mg of chicken feet hydrolysate (CFH)/kg body weight, diluted in a sugary solution (sucrose/water; 1:1 *w/v*) or with the vehicle (VH group), composed of 400 mg/kg of maltodextrin diluted in the same sugary solution. After three weeks of treatment, animals were sacrificed and tissues collected. Four or five days before sacrifice, indirect calorimetry was performed on eight random animals per group for 24 h. One day prior to sacrifice, the fat mass and lean mass of the other eight animals per group were analyzed by quantitative magnetic resonance (QMR).

A total of 32 five-week-old male Wistar rats (Charles River Laboratories, Barcelona, Spain) were housed two per cage at 22 °C and 55% humidity with a light/dark period of 12 h and with free access to food and water (Figure 1). After an adaptation period of four days, animals were fed a cafeteria diet (CAF) ad libitum, consisting of bacon, biscuits with pâté and cream cheese, ensaïmada (sweetened pastry), carrots, semihard cheese, and sweetened milk (20% sucrose *w/v*), in addition to the standard chow pellets (Panlab, Barcelona, Spain) for 11 weeks. CAF diet is a diet model based on appetizing ingredients rich in saturated fats and simple carbohydrates that induce voluntary hyperphagia, developing the main features of metabolic syndrome and obesity [27]. For the last three weeks, animals were randomly distributed in two groups (*n* = 16) and orally supplemented with a mix (mix group) of natural ingredients [14], composed of 25 mg GSPE/kg body weight, 100 mg CLA/kg body weight, 100 mg anthocyanins/kg body weight, and 55 mg of CFH/kg body weight, diluted in a sugary solution (sucrose/water; 1:1 *w/v*) or with the vehicle (VH group), composed of 400 mg/kg of maltodextrin diluted in the same sugary solution. The doses were chosen according to the previous results of our group and other published studies. The equivalent daily dose in a human of 65 kg would be 1 g of anthocyanins, 1 g of CLA, 0.26.g of GSPE, and 0.58 g of CFH [28]. Due to the palatability of the treatment, animals voluntarily drank their daily dose without requiring oral gavage.

The body weight and food intake of the animals were recorded once each week. One day prior to sacrifice, the fat mass and lean mass of eight animals per group were analyzed by quantitative magnetic resonance (QMR) using an EchoMRI-700 ^TM^ (Echo Medical Systems, LLC. Houston, TX, USA) without anesthesia. After the eleventh week of the experiment, all animals were fasted for three hours and sacrificed by decapitation. The IWAT and retroperitoneal WAT (RWAT), epidydimal WAT (EWAT), and mesenteric WAT (MWAT), as well as interscapular BAT, were rapidly removed after death, weighed, frozen in liquid nitrogen, and stored at −80 °C until further analysis.

The percentage of fat and lean mass and that of the different adipose tissue deposits were calculated as a percentage of total body weight. The adiposity index was computed as the sum of EWAT, MWAT, IWAT, and RWAT depot weights and expressed as a percentage of total body weight.

The Animal Ethics Committee of University Rovira i Virgili (Tarragona, Spain) approved all the procedures (reference number 7959, 23/11/2016 by Generalitat de Catalunya), and the guidelines for the use and care of laboratory animals of the university were followed. All of the abovementioned experiments were performed as authorized (European Directive 86/609/CEE and Royal Decree 223/1988 of the Spanish Ministry of Agriculture, Fisheries, and Food, Madrid, Spain).

### 2.3. Indirect Calorimetry

Indirect calorimetry was performed on eight random animals per group for 24 h, four or five days before sacrifice using an Oxylet Pro System (Panlab, Barcelona, Spain) and the Metabolism 2.1.02 (Panlab, Cornellà, Spain) software program as explained previously [29]. In short, fat and glucose oxidation were calculated with the oxygen consumption (VO2) and carbon dioxide production (VCO2) values given by the Oxylet LE 405 gas analyzer (PANLAB). At each time point, the program Metabolism 2.1.02 (PANLAB, Barcelona, Spain) calculated the respiratory quotient (RQ) as the VCO2/VO2 ratio. Using the stoichiometric equations of Frayn [30] and assuming a nitrogen excretion rate (n) of 135 μg kg^−1^ min^−1^ [31], we used the formula (g min^−1^) = 4.55 × VCO2 − 3.21 × VO2 − 2.87 n for the oxidation of carbohydrates and the formula (g min^−1^) = 1.67 × VO2 − 1.67 × VCO2 − 1.92 n for the oxidation of fat. To obtain the EE from fat and carbohydrate in kJ min^−1^, the fat and carbohydrate rates were multiplied by 37 and 16, respectively, using the Atwater general conversion factor [32]. Due to the differences in metabolic activity in rodents during day and night, we separated the results of both light cycles to better appreciate differences between groups [33,34].

### 2.4. Cell Culture and Treatment

Undifferentiated 3T3-L1 preadipocytes were propagated and induced to differentiate [35]. Cells were treated throughout the differentiation process, with the treatments added into the medium with each medium change. The following treatments were tested: 10, 25, 50, 75, or 100 µg/mL of GSPE; 10, 50, 100, 250, or 500 µg/mL of CLA; and 2, 10, 20, 50, or 100 µg/mL of anthocyanins dissolved in 0.1% dimethyl sulfoxide (DMSO) and phosphate-buffered saline (PBS). Cell viability was assessed with neutral red, and all of the cell experiments were repeated three times for each group. The triglyceride content of cells after eight days of differentiation was assessed with Oil Red O staining [36] with the following method. 3T3-L1 cells were fixed with a 10% formalin solution and stained with an Oil Red O solution prepared with 0.5% Oil Red O dye (Sigma-Aldrich, Madrid, Spain) in 100% isopropanol, diluted with water (six parts Oil red O isopropanol solution/four parts water). After an incubation of 1 h, the dye was extracted from the cells with isopropanol and transferred to a 96-well plate. Optical density was measured with an Eon TM high-performance microplate spectrophotometer (Biotek Instruments, Inc., Winooski, VT, USA) at 510 nm.

### 2.5. RNA Extraction and Quantification by Real-Time qRT-PCR

Total RNA from IWAT, EWAT, RWAT, and BAT tissues was extracted using Trizol^®^ reagent (Ambion, Life Technologies, Uppsala, Sweden) following the manufacturer’s instructions. The RNA yield was quantified in a Nanodrop ND-1000 spectrophotometer (NanoDrop Technologies, Wilmington, DE, USA).

A total of 0.5 µg of total RNA was reverse transcribed using a high-capacity cDNA reverse transcription kit (Applied Biosystems, Madrid, Spain) in a multigene thermal cycler (Labnet, Madrid, Spain). For qPCR, the CFX96 real-time system C1000 touch thermal cycler (Bio-Rad, Barcelona, Spain) with the iTaq™ Universal SYBR^®^ Green Supermix (Bio-Rad, Barcelona, Spain) was used.

Gene expression levels in IWAT, EWAT, RWAT, and BAT tissues and 3T3-L1 were assessed for the genes indicated in (Table S2). The primers for the different genes are also described in Table S2 and were obtained from Biomers.net (Ulm, Germany). The relative expression of each mRNA was calculated as a percentage of the vehicle group using the 2^−∆∆Ct^ method [37], with *Ppia*, *Actb*, and *Hprt1* as the reference genes. Each PCR was performed at least in duplicate.

### 2.6. Histology Analysis

For histological analyses, frozen IWAT samples were thawed and fixed in 4% formaldehyde and processed as explained previously [38].

Sections were observed and acquired at 10× magnification using AxioVision Zeiss Imaging software (Carl Zeiss Iberia, S.L., Madrid, Spain). The area and number of adipocytes were measured using the open source software Adiposoft (CIMA, University of Navarra, Spain). Four fields per sample and six samples from each group were measured, and the area, total adipocyte number, and adipocyte size frequencies were calculated as explained previously [38].

### 2.7. Western Blot Analysis

Fasn protein content in 3T3-L1 was determined by Western blot. Cells were homogenized in radioimmunoprecipitation assay (RIPA) lysis buffer. The protein content was quantified using a BCA protein assay kit (Pierce, Rockford, IL, USA), and the anti-Fasn antibody was acquired from Abcam (Cambridge, United Kingdom). The Western blot and protein level quantification were performed as described previously [38].

### 2.8. Stromal Vascular Fraction Extraction

Immediately after IWAT was dissected and washed in Krebs–Henseleit (K–H) pH 7.4 buffer. The tissue was minced and digested in incubation buffer (1 mM Cl2Ca, 3% Bovine Serum Albumin, 5 mM glucose) and type 1 collagenase from *Clostridium histolyticum* (Worthington, Lakewood, NJ, USA) for 1 h at 37 °C. The digested tissue was filtered with a 200 µm cell strainer, and washing buffer (0.5 mM Cl2Ca, 3% BSA in K–H buffer) was added, followed by centrifugation at 400 g for 5 min. Erythrocytes were lysed with a red blood cell lysis buffer (0.154 M NH4Cl, 10 mM KHCO3, and EDTA in distilled water). Afterward, cells were washed with washing buffer and centrifuged for 15 min at 400 g. The pellet was frozen in RLT buffer from an RNeasy ^®^ Mini kit (Qiagen, Barcelona, Spain).

### 2.9. Statistical Analysis

The software SPSS (SPSS, Chicago, IL, USA) was used for statistical analysis. Data are expressed as mean ± SEM or box plots, and significant differences were analyzed by unpaired Student’s *t*-test. Cell culture results were analyzed with one-way ANOVA followed by Duncan’s post hoc test. A *p*-value ≤ 0.05 was considered statistically significant.

## 3. Results

### 3.1. Biometric Measures

Rats supplemented with the mix of ingredients showed a significant 7.99% reduction in weight and a significant 42.4% reduction in body weight gain compared to the VH group (Table 1). After two weeks of treatment, animals started to show a significant decrease in body weight compared to the vehicle group (Figure S1). Fat (%) was reduced by 15.3%, while fat (g) showed a tendency to also be reduced in the mix group (*p* = 0.095). EWAT (g) and IWAT (g) depots showed a significant 25.8 and 21.7% reduction, respectively. Similarly, EWAT (%) also showed a significant reduction, while RWAT and MWAT showed no differences compared to the VH group. RWAT (g) and IWAT (%) showed a tendency to be reduced by the treatment (*p* = 0.069 and *p* = 0.086, respectively). The adiposity index was significantly reduced by 12.34%, and lean mass (%) was significantly increased in the mix group compared to the VH group (Table 1).

### 3.2. Indirect Calorimetry

**Figure 2:**
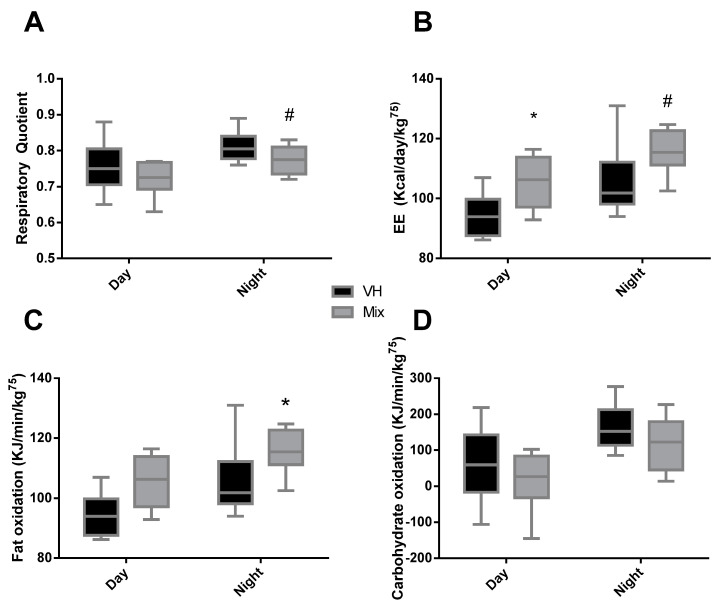
Respiratory quotient (RQ) (**A**), energy expenditure (EE) (**B**), fat oxidation (**C**), and carbohydrate oxidation (**D**) during day and night, measured during 24 h four or five days before sacrifice, of Wistar rats fed a CAF diet for 11 weeks and supplemented with the vehicle or a mix of ingredients containing GSPE, anthocyanins from bilberry and blackcurrant, CLA, and CFH during the last three weeks. Box plots represent the median and Tukey whiskers (*n* = 8), and both groups were compared with Student’s *t*-test (* *p* < 0.05, ^#^ *p* < 0.1).

The group treated with the mix of ingredients significantly increased the EE during the day and showed a tendency (*p* = 0.070) to increase it during the night as well. Additionally, we observed a significant increase in fat oxidation during the night in the mix group, while no differences were observed during the day. Carbohydrate oxidation and RQ showed no significant differences; however, at night, the RQ of the mix group showed a tendency to be lower (*p* = 0.1) (Figure 2).

### 3.3. Gene Expression in IWAT, EWAT, RWAT, BAT, and SVF

**Figure 3:**
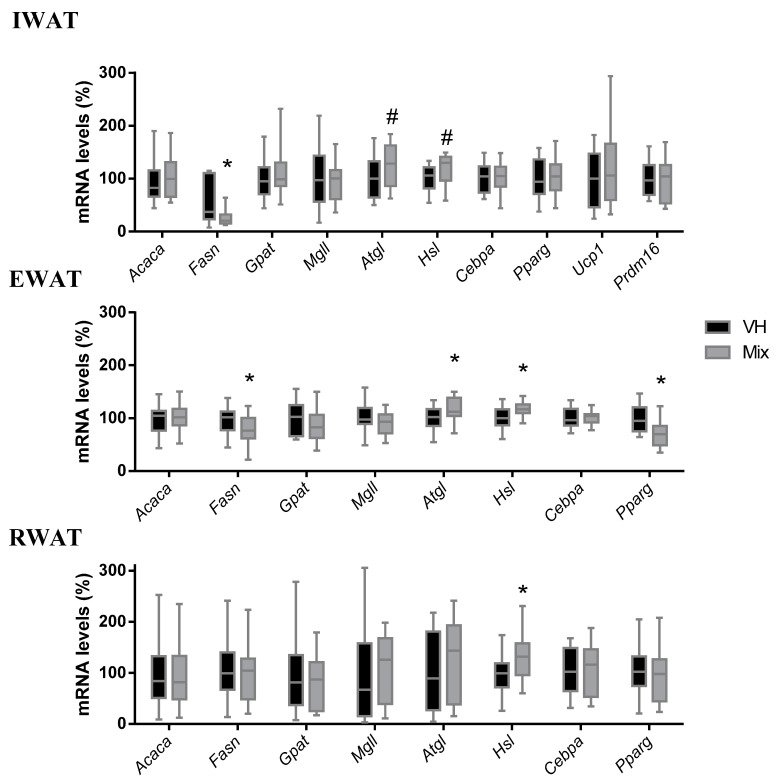
Expression of genes related to lipogenesis, lipolysis, adipogenesis, and thermogenesis in the IWAT, EWAT, and RWAT of Wistar rats fed a CAF diet for 11 weeks and supplemented with the vehicle or a mix of ingredients containing GSPE, anthocyanins from bilberry and blackcurrant, CLA, and CFH during the last three weeks. Data are presented as the ratios of gene expression, relative to β-actin, *Ppia*, and *Hprt* and expressed as a percentage of the VH group, set at 100%. Box plots represent the median and Tukey whiskers (*n* = 16), and data were compared with Student’s *t*-test (* *p* < 0.05, ^#^ *p* < 0.1).

In EWAT, mix treatment significantly reduced the gene expression of *Fasn* and *Pparγ*, while it increased the expression of *Atgl* and *Hsl* genes compared to the VH. In IWAT, the mix treatment highly reduced *Fasn* gene expression, while it showed a tendency to upregulate the gene expression of *Atgl* and *Hsl* (*p* = 0.078 and *p* = 0.055, respectively). Additionally, in the IWAT, we analyzed the gene expression of *Ucp1* and *Prdm16*, but their expression was unaffected by the treatment. RWAT showed a significant increase in *Hsl* gene expression in the mix group compared to the VH group, and no changes were observed in any of the other genes analyzed (Figure 3).

The SVF extraction protocol is lengthy and must be done immediately after killing the animal; for this reason, only SVF from the IWAT was extracted and analyzed. SVF showed no differences in the expression level of any of the genes analyzed (Table S3).

In BAT, although a slight increase in *Ucp1* and *Prdm16* was observed in the mix group, no significant differences among the groups were found (Table S4).

### 3.4. Histology of the IWAT

**Figure 4:**
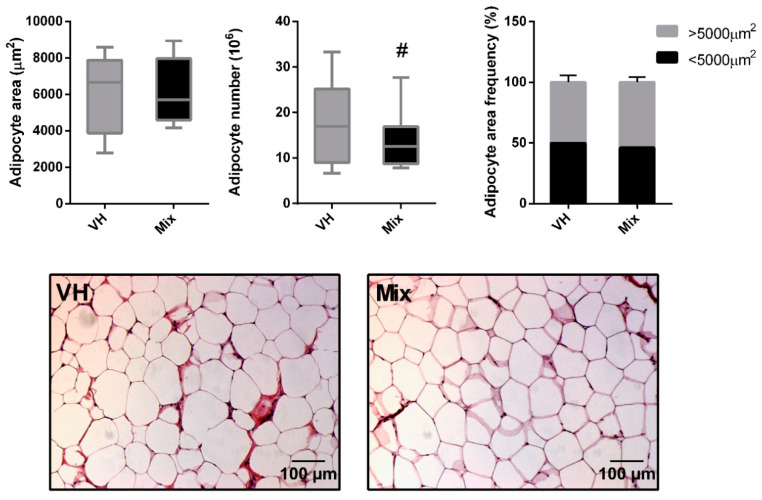
Adipocyte area, adipocyte number, and adipocyte area frequencies in IWAT of rats fed a CAF diet for 11 weeks and supplemented with the vehicle or a mix of ingredients containing GSPE, anthocyanins from bilberry and blackcurrant, CLA, and CFH during the last three weeks. Representative pictures of both CAF groups are shown. For frequencies, adipocytes were distributed in two groups depending on their areas (<5000 or >5000 µm^2^). Box plots represent the median and Tukey whiskers (*n* = 16), and statistical significance was analyzed via Student’s *t*-test (^#^ *p* < 0.1).

The IWAT depot showed no differences at the histological level in terms of adipocyte area, adipocyte number, or adipocyte area frequency between the study groups (Figure 4). Although not significant (*p* = 0.10), the total number of adipocytes was slightly reduced in the mix group compared with the VH group.

### 3.5. 3T3-L1 Results

**Figure 5:**
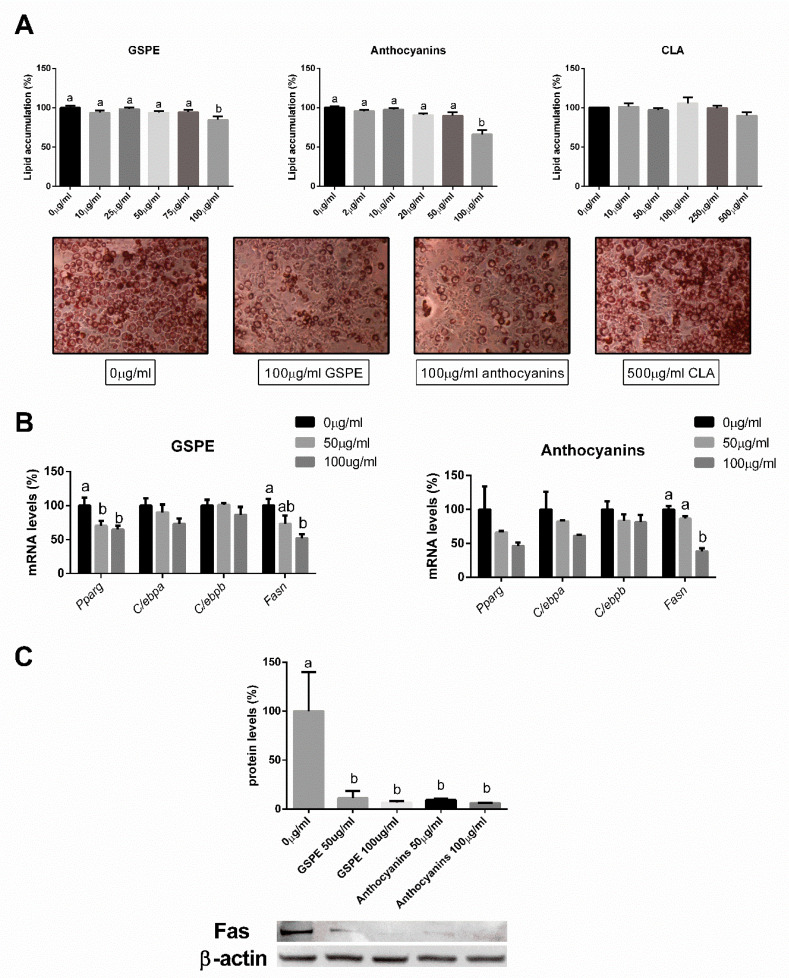
Effects of GSPE, anthocyanins, and CLA on lipid accumulation measured with Oil Red O (**A**), effects of GSPE and anthocyanins on mRNA levels of adipogenesis and lipogenesis genes (**B**), and protein levels of *Fasn* measured with Western blot of differentiated 3T3-L1 cells treated with various concentrations during the differentiation phase (**C**). The data are expressed as the mean ± SEM (*n* = 16) of three independent experiments, and differences among groups are measured with one-way ANOVA followed by Duncan’s post hoc test. Mean values with unlike letters have significant difference among groups.

Results showed that 100 µg/mL of GSPE and 100 µg/mL of anthocyanins significantly reduced lipid accumulation in 3T3-L1 cells when compared to the untreated group (Figure 5A). CLA showed no changes in lipid accumulation; therefore, its gene and protein expression profiles were not quantified.

*Pparγ* gene expression was reduced by treatment with 50 and 100 µg/mL of GSPE, while *Fasn* levels were reduced by the 100 µg/mL GSPE treatment. Anthocyanins at a 100 µg/mL concentration sharply reduced *Fasn* RNA levels. No changes were observed in *C/ebpα* and *C/ebpβ* (Figure 5B) between groups.

The same concentrations of GSPE and anthocyanins used for the gene expression analysis were used to measure protein concentration of *Fasn*. All four concentrations of GSPE and anthocyanins greatly reduced *Fasn* protein levels (Figure 5C) when compared with the untreated group.

## 4. Discussion

The results of previous studies realized by our research group and other researchers suggest that GSPE, anthocyanins, CLA, and CFH could have a positive effect by counteracting the different ailments related to obesity [15,16,17,18,19]. The objective of this study was to evaluate the effect of the combination of these four ingredients in the form of a mix on the expanded adipose tissue of obese rats.

Our results showed that rats treated with the mix for three weeks had a clear reduction in weight and body weight gain compared to the untreated group and that this effect was accompanied by a reduction in fat mass. Furthermore, the treatment did not alter lean mass but only altered its percentage over body weight, indicating that it was caused by decreased levels of fat. Several studies have shown that weight loss achieved by caloric restriction decreases lean mass, which is an undesirable effect that can be attenuated with exercise [39].

Specifically, both IWAT and EWAT mass depots were greatly reduced by the treatment, while RWAT showed a smaller and nonsignificant reduction and MWAT was unaffected. The heterogeneous effect observed by the mix treatment on the reduction in adipose depot mass is probably explained by the described intrinsic differences among fat deposits. In this sense, studies have found that subcutaneous and visceral fat depots are morphologically and functionally different [40,41]. On the other hand, the BAT also stores fat and has been shown to increase in weight during a HFD [42,43,44,45]; thus, as shown in this and other studies in rodents, body weight reduction is accompanied by decreased BAT weight [46,47].

Our results indicate both an increase in EE and greater lipid utilization in the mix group compared to the control animals, which could explain the decrease in fat accretion. According to recent investigations by Guo and Ling [22] and Salvadó et al. [21], the effects of anthocyanins and proanthocyanidins do not seem to be attributed to an increase in EE in obesity. In a recent study, even though treatment with 0.5 g/kg body weight of GSPE showed an increase in EE in lean rats, a higher dose in the same study showed no differences compared to the control, suggesting that only specific doses are able to affect this parameter [48]. Other studies with GSPE and anthocyanins have reported increased levels of fat oxidation [22,29,49]. In contrast, studies on obese mice supplemented with a particular CLA isomer have shown increased EE and fat oxidation, resulting in fat loss [18]. Additionally, Terpstra et al. reported increased EE in mice fed an obesogenic diet supplemented with CLA [50]. We principally attribute the observed differences in EE to the CLA; however, its effects on fat oxidation are probably amplified by supplementation with the other ingredients.

The mix sharply decreased *Fasn* gene expression levels and showed a tendency to upregulate *Atgl* and *Hsl* in the IWAT. A clearer effect was observed in EWAT, where *Ppar*γ levels were also decreased, while in the RWAT, which showed more variability among genes, only *Hsl* was increased. These results are in accordance with the differences observed in the weights of each adipose depot and suggest that IWAT and EWAT depots are more influenced by the treatment than the RWAT. The gene expression results further agree with the greater fat oxidation observed in the calorimetry results as both *Atgl* and *Hsl* play an important role in the lipolysis process [51]. Notably, *Atgl* and *Hsl* mRNAs have been found to be increased in the WAT of lean Zucker rats compared to their obese counterparts [52]. Moreover, overexpression of *Atgl* in transgenic mice has been shown to reduce diet-induced obesity and promote fatty acid oxidation [53]. Altogether, these data suggest that increased *Atgl* and *Hsl* levels are associated with a healthier and leaner phenotype. Additionally, GSPE treatment in obese hamsters has also shown to increase *Hsl* and *Atgl* in RWAT depots, which supports our results [15]. The downregulation shown in our study of *Fasn* and *Pparγ*, which are key genes of lipogenesis and adipogenesis [54], respectively, together with data from other authors, suggest an inhibition of lipogenesis in IWAT and EWAT, while adipogenesis is also affected but only in the EWAT. Altogether, the changes observed in the gene expression, particularly in the IWAT and EWAT depots, agrees with the clear effects of the mix on fat accretion and with the increase in lipid oxidation observed with indirect calorimetry.

We decided to test the individual ingredients on the 3T3-L1 cell line to complement the reported effects of the mix in vivo and to formulate a hypothesis of the ingredients that might have more effect on the adipose tissue. Our experiments with 3T3-L1 cell culture supported a potential lipogenic and adipogenic inhibition effect of GSPE and anthocyanins through a reduction in lipid accumulation, inhibition of key genes such as *Fasn* and *Pparγ*, and reduction of *Fasn* protein levels. Our results in 3T3-L1 are in accordance with other authors who have used these cells. Treatment with 140 µg/mL GSPE at the onset of differentiation reduced adipogenesis markers [7], while an inhibition of lipid content and adipogenic transcription factors was observed with a concentration of 100 µg/mL of anthocyanins extracted from *Vitis coignetiae* [8]. Unexpectedly, we obtained no differences in the lipid content of cells with CLA treatment, while other authors showed that 10 mg/L of CLA isomers, consisting principally of 41% c9, t11 and 44% t10, c12, inhibited proliferation of 3T3-L1 preadipocytes while promoting lipogenesis [55]. Other experiments have shown that the effects of CLA are truly influenced by the isomer used and the stage at which differentiation is applied [9,56,57]. Altogether, it appears that in cell culture experiments, the isomer t10, c12 has a more evident effect on inhibiting cell differentiation, while the isomer c9, t11 might increase lipid accumulation and promote preadipocyte differentiation. However, it must be taken into consideration that the literature is sometimes contradictory. We conclude that the lack of effect of our CLA treatment on the 3T3-L1 cell line might be related to the particular mix of isomers we used, including the other fatty acids in the mixture, as described in Table S1.

According to our in vitro results, the effects observed specifically in the rat WAT are probably related to GSPE and anthocyanins. In fact, Pascual-Serrano et al. found that diet-induced obese rats supplemented with a dose of 25 mg/kg body weight of GSPE for three weeks did not show reduced body weight or fat; however, the treatment reduced the size of the adipocytes and increased their number, which ameliorated hyperglycemia and dyslipidemia [16]. It has also been reported that hamsters fed a HFD for 30 days and supplemented with 25 mg/kg body weight of GSPE for the last 15 days can show a reduction in the adiposity index and the weight of fat depots, accompanied by an upregulation of β-oxidation in the RWAT [15]. Anthocyanins have also been reported to be useful for the prevention and treatment of obesity [22]. Focusing on the WAT, Wu et al. showed that an eight-week treatment with 200 mg/kg body weight of anthocyanins reduced 30.19% of the weight of the EWAT of mice fed a HFD for 16 weeks compared to that of the control group, without changes in food intake [17]. The mechanisms they propose include a decrease in food efficiency and suppression of genes related to fatty acid and TAG synthesis [17]. More specifically, human studies have shown the effectiveness of the same anthocyanin extract that we used in improving the serum profile in dyslipidemic subjects [25] and the prevention of insulin resistance in diabetic patients [58]; however, no specific effects in fat have been reported to our knowledge. Altogether, our results confirm a beneficial effect of GSPE and anthocyanins on fat accumulation and suggest that, in in vivo experiments, these ingredients have a synergic effect because a clear effect on body fat mass loss is observed.

CFH is a hydrolysate made from chicken feet following the method described by Bravo et al., and it has been found to be useful for the treatment of hypertension in rats; however, there have not been any studies reporting differences in body weight or fat content [19,26]. Thus, the purpose of including CFH in the mix of ingredients was to reduce hypertension, a measurement that will be evaluated in future publications and was not the objective of this study.

To further explain the mechanisms by which the mix affected the fat mass depots, we studied the browning process of the WAT. We focused on the IWAT depot because of the evidence showing CLA can increase thermogenesis of the subcutaneous adipose tissue in obese mice [18,59] and because it has been described that the IWAT or subcutaneous WAT expresses higher levels of browning genes, such as *Ucp1* and *Prdm16*, compared to other white adipose tissues [60]. Additionally, other studies in obese mice supplemented with CLA have reported increased browning and thermogenesis in other WAT deposits [61,62]. However, in our study, we did not observe any changes in *Ucp1* and *Prdm16* gene expression, which might imply that thermogenesis or browning in the IWAT was not promoted by our treatment. In this sense, to our knowledge, there are no published results showing a browning effect of GSPE or anthocyanidins treatments in the WAT.

The subcutaneous WAT in humans has been reported to possess a larger pool of preadipocytes than other WAT depots [41]. Thus, we expected that, in case the treatment was affecting the adipocyte differentiation, we would be able to detect changes in gene expression in the SVF. However, this was not the case, and in agreement with the *Pparγ* levels, adipogenesis did not appear to be altered in the SVF of the IWAT, suggesting that the treatment affected the tissue in another way. For this reason, we studied the morphology of the IWAT through histology and observed a slight tendency of a decrease in the total number of adipocytes; however, no differences in adipocyte area were observed. Even though we did not obtain strong evidence, these data together seem to indicate that the reduction in fat content by the treatment is caused by a reduction in the number of adipocytes, without affecting their area. We postulate that even though differences at a biometrical level and in terms of gene expression in the IWAT were obvious, histology only focuses on a small part of the deposit, and it might thus not be representative of the whole tissue.

Because of the increased EE and lipid oxidation observed in rats treated with the mix, we studied the gene expression levels of BAT, which is known to metabolize lipids in the organism to dissipate energy, increasing EE in the organism [10]. However, no differences were observed in the gene expression of key genes involved in lipid uptake, β-oxidation, and thermogenesis, and thus, it appears that the BAT is not implicated in the diminished fat accretion or the increased EE of the mix group. Thus, future studies should also focus on other highly oxidative tissues, such as the liver and the skeletal muscle, as being responsible for the observed increase in EE.

## 5. Conclusions

In conclusion, the mix of natural ingredients used in this study administered to rats fed a CAF diet was able to decrease fat accretion after three weeks of treatment, increase EE, increase fat oxidation, and modify the gene expression of key genes in lipid metabolism of the adipose tissue. Moreover, the fat loss achieved by the treatment preserved lean mass, the loss of which has a detrimental effect that is sometimes associated with weight loss. Although further research is needed on the mix of ingredients and the individual compounds, also taking into account other important parameters such as sex or age, we observed a clear and strong effect of the mix on body weight reduction. These results suggest that this mix of bioactive compounds could be useful for the treatment of obesity in humans and indicate that the synergy of the ingredients improves the beneficial effects in the WAT that has been observed in other studies with the individual ingredients. Having confirmed our hypothesis with the mix of ingredients, in future work, we intent to delve further into the mechanisms of action of the individual components of the mix and to fully comprehend the advantages of using this mix over the individual compounds. Moreover, due to the known metabolic modification of ingested polyphenols and other compounds caused by digestion and the microbiota, we want to research the effects of the metabolites found in the plasma after supplementation with the mix and their effects on the adipose tissue through cell culture studies.

## Figures and Tables

**Table 1:** Biometric measures of rats fed a cafeteria (CAF) diet treated with a mix of natural bioactive ingredients or the vehicle.

	VH	Mix	*p*-Value
Initial weight (g)	172.60 ± 4.43	172.80 ± 5.30	0.976
Weight before treatment (g)	504.50 ± 11.98	481.90 ± 7.96	0.123
Weight after treatment (g)	552.27 ± 14.17	508.13 ± 9.13 *	0.013
Weight gain before treatment (%)	193.82 ± 7.40	181.84 ± 7.65	0.270
Weight gain during treatment (%)	9.41 ± 0.52	5.42 ± 0.54 *	<0.001
Accumulated caloric intake (Kcal)	4305.43 ± 245.68	3826.15 ± 220.22	0.150
Fat (g)	132.43 ± 6.93	109.53 ± 10.76 ^#^	0.095
Lean (g)	366.64 ± 7.19	373.64 ± 6.14	0.471
Fat (%)	25.01 ± 0.91	21.19 ± 1.59 *	0.054
Lean (%)	69.58 ± 1.05	73.41 ± 1.25 *	0.035
EWAT (g)	26.09 ± 1.83	19.37 ± 1.37 *	0.006
IWAT (g)	10.21 ± 0.68	7.99 ± 0.57 *	0.019
RWAT (g)	27.13 ± 1.84	22.75 ± 1.43 ^#^	0.068
MWAT (g)	12.49 ± 1.28	10.29 ± 1.00	0.182
BAT (g)	1.064 ± 0.07	0.869 ± 0.037 *	0.017
Adiposity Index (%)	13.61 ± 0.65	11.93 ± 0.52 *	0.050
EWAT (%)	4.68 ± 0.25	3.78 ± 0.22 *	0.012
IWAT (%)	1.84 ± 0.1	1.57 ± 0.11 ^#^	0.086
RWAT (%)	4.87 ± 0.25	4.45 ± 0.24	0.233
MWAT (%)	2.22 ± 0.19	1.99 ± 0.16	0.351
BAT (%)	0.192 ± 0.011	0.171 ± 0.007	0.123

Wistar rats fed a CAF diet for 11 weeks and supplemented with the vehicle (VH) or a mix of ingredients containing GSPE, anthocyanins from bilberry and blackcurrant, CLA, and CFH during the last three weeks. Weight gain and accumulated caloric intake were calculated with the data obtained at the start and end of the treatment. The adiposity index was computed as the sum of EWAT, MWAT, IWAT, and RWAT depot weights and expressed as a percentage of total body weight. BAT, interscapular brown adipose tissue; EWAT, epididymal white adipose tissue; MWAT, mesenteric white adipose tissue; IWAT, inguinal white adipose tissue; and RWAT, retroperitoneal white adipose tissue. Data are presented as the mean ± SEM (*n* = 16), and both groups were compared with Student’s *t*-test (* *p* < 0.05, ^#^ *p* < 0.1).

## Supplementary Materials

The following are available online at https://www.mdpi.com/2072-6643/12/11/3251/s1, Figure S1: Body weight increase in rats fed a CAF diet with or without the mix treatment, Table S1: Fatty acid profile of the conjugated linoleic acid ingredient, Table S2: Primers for the qPCR analysis, Table S3: Gene expression levels of the SVF of rats fed a CAF diet with or without the mix treatment, Table S4: Gene expression levels in the BAT of rats fed a CAF diet with or without the mix treatment.
