# Supplementary material for: A Mix of Natural Bioactive Compounds Reduces Fat Accumulation and Modulates Gene Expression in the Adipose Tissue of Obese Rats Fed a Cafeteria Diet
Source: Nutrients. 2020 Oct 23;12(11):3251. doi: 10.3390/nu12113251 (PMC7690777; doi:10.3390/nu12113251)
Supplement: Supplementary file 1 [file nutrients-12-03251-s001.pdf]

Supplementary Materials

Table S1. Fatty acid profile of the Conjugated Linoleic Acid ingredient.

	%
C16:O	1.5
C18:O	2.9
C18:1, c9	13.2
C18:2, c9c12	0.4
CLA, total	78.5
CLA c9, t11	38.4
CLA t10, c12	37.9
CLA c,c isomers	1.1
CLA t,t isomers	1
CLA t8, c10	<0.1
CLA c11, t13	<0.1
Triglyceride	92.1
Diglyceride	6.5
Monoglyceride	0
Free fatty acids	0.5
Glycerol	<0.1

Data obtained from the manufacturer (Tonalin® CLA).

Table S2. Primers for the Q-PCR analysis.

	**Forward (5′...3′)**	**Reverse (5′...3′)**
Rattus norvegicus		
Hprt	TCCCAGCGTCGTGATTAGTGA	CCTTCATGACATCTCGAGCAAG
Actb	GCAGGAGTACGATGAGTCCG	ACGCAGCTCAGTAACAGTCC
Ppia	CTTCGAGCTGTTTGCAGACAA	AAGTCACCACCCTGGCACATG
Acacα	GCGGCTCTGGAGGTATATGT	TCTGTTTAGCGTGGGGATGT
Atgl	GAAGACCCTGCCTGCTGATT	CACATAGCGCACCCCTTGAA
Fasn	TAAGCGGTCTGGAAAGCTGA	CACCAGTGTTTGTTCCTCGG
Gpat	GAATACAGCCTTGGCCGATG	GAGGCGTGCATGAATAGCAA
Hsl	AGTTCCCTCTTTACGGGTGG	GCTTGGGGTCAGAGGTTAGT
Prdm16	GTTCTGCGTGGATGCCAATC	TGGCGAGGTTTTGGTCATCA
Cebpα	TGTACTGTATGTCGCCAGCC	TGGTTTAGCATAGACGCGCA
Mgll	ATCATCCCCGAGTCAGGACA	TGACTCCCCTAGACCACGAG
Ucp1	GGTACCCACATCAGGCAACA	TCTGCTAGGCAGGCAGAAAC
Lpl	GGCCCAGCAACATTATCCAG	ACTCAAAGTTAGGCCCAGCT
Had	ATCGTGAACCGTCTCTTGGT	AGGACTGGGCTGAAATAAGG
Cpt1b	GCAAACTGGACCGAGAAGAG	CCTTGAAGAAGCGACCTTTG
Pparα	CGGCGTTGAAAACAAGGAGG	TTGGGTTCCATGATGTCGCA
Fatp1	CTACCACTCAGCAGGGAACA	GCGGCATATTTCACCGATGT
Cd36	CAGTGCAGAAACAGTGGTTGTCT	TGACATTTGCAGGTCCATCTATG
Pparγ	AGGGCGATCTTGACAGGAAA	CGAAACTGGCACCCTTGAAA
Pref-1	CCCGGAGAAGATCGACATGA	TTGTCACACAGCAACACGAG
Mus musculus		
Hprt	GGCTTACCTCACTGCTTTCCG	TAATCACGACGCTGGGACTGC
Actb	GCAGGAGTACGATGAGTCCG	ACGCAGCTCAGTAACAGTCC
Ppia	CTTCGAGCTGTTTGCAGACAA	AAGTCACCACCCTGGCACATG
Fasn	TTCGGTGTATCCTGCTGTCC	TGGGCTTGTCCTGCTCTAAC
Cebpα	GGTGGACAAGAACAGCAACGA	CGTTGCGTTGTTTGGCTTTATC
Cebpβ	TGCGGGGTTGTTGATGTTTT	TGCTCGAAACGGAAAAGGTT
Pparγ	AGGGCGATCTTGACAGGAAA	CGAAACTGGCACCCTTGAAA

Hypoxanthine-guanine phosphoribosyltransferase (*Hprt*), Actin beta (*Actb*), Peptidylprolyl Isomerase A (*Ppia*), acetyl-CoA carboxylase alpha (*Acacα*), adipose triglyceride lipase (Atgl), fatty acid synthase (*Fasn*), glycerol-3-phosphate acyltransferase (*Gpat*), hormone-sensitive lipase (*Hsl*), PR domain

containing 16 (*Prdm16*), CCAAT/enhancer-binding protein alpha (*C/ebpα*), monoglyceride lipase (*Mgll*), uncoupling protein 1 (*Ucp1*), lipoprotein lipase (*Lpl*), hydroxyacyl-CoA dehydrogenase (*Had*), carnitine palmitoyltransferase 1B (*CPT1b*), peroxisome proliferator-activated receptor alpha (*Pparα*), fatty acid transport protein 1 (*Fatp1*), cluster of differentiation 36 (*Cd36*), peroxisome proliferator-activated receptor gamma (*Pparγ*), Preadipocyte Factor-1 (Pref-1).

Table S3. Gene expression levels of the SVF of rats fed a CAF diet with or withouth de mix treatment.

	VH	Mix
Acacα	100 ± 25.23	110.02 ± 22.66
C/ebpα	100 ± 15.05	104.58 ± 15.2
Pref-1	100 ± 32.62	80.02 ± 7.68
Lpl	100 ± 22.03	136.17 ± 31.84
Pparγ	100 ± 13.63	106.56 ± 22.68
Fasn	100 ± 42.53	207.87 ± 97.94

Expression of genes related with lipogenesis, lipolysis and adipogenesis in the SVF of the IWAT of Wistar rats fed a CAF diet and treated either with vehicle (VH) or the mix (mix). Data is presented as the ratios of gene expression, relative to β-actin, ppia and hprt and expressed as a percentage of the VH group, set at 100%. Results are presented as the mean ± SEM and data compared with Student's *t*-test (* $p < 0.05$, # $p < 0.1$).

Table S4. Gene expression levels in the BAT of rats fed a CAF diet with or withouth de mix treatment.

	VH	Mix
Ucp1	100 ± 8.34	118.81 ± 9.53
Prdm16	100 ± 6.32	113.15 ± 4.97
Pparα	100 ± 7.94	113.59 ± 7.52

Expression of genes related with the activity of the BAT of Wistar rats fed a CAF diet and treated either with vehicle (VH) or the mix (mix). Data is presented as the ratios of gene expression, relative to *β-actin*, *Ppia* and *Hprt* and expressed as a percentage of the VH group, set at 100%. Results are presented as the mean ± SEM and data compared with Student's *t*-test (* $p < 0.05$, # $p < 0.1$).

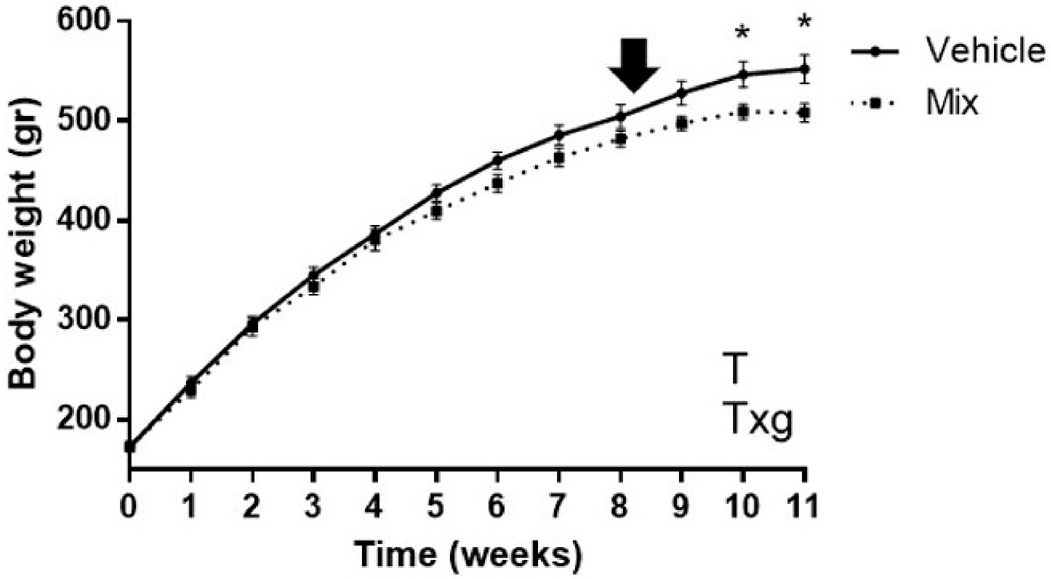

Figure S1. Body weight increase of Wistar rats fed a CAF diet for eleven weeks and supplemented with the vehicle (VH) or a mix of ingredients (Mix) containing grape-seed proanthocyanidin extract, anthocyanins from bilberry and blackcurrant, conjugated linoleic acid and a chicken feet hydrolysate during the last three weeks of experiment. The arrow indicates the starting point of the treatment, week 9. Data are presented as the mean ± SEM (n = 16), and both groups are compared with a repeated-measures ANOVA comparison. T denotes a significant effect of time ($p < 0.005$) and TxG

denotes the significant interaction of time and group ($p < 0.005$). Differences in a particular time point were assessed with Bonferroni's multiple comparison test (* $p < 0.05$).
